# Acute Ethanol Challenge Differentially Regulates Expression of Growth Factors and miRNA Expression Profile of Whole Tissue of the Dorsal Hippocampus

**DOI:** 10.3389/fnins.2022.884197

**Published:** 2022-05-30

**Authors:** Thaddeus M. Barney, Andrew S. Vore, Terrence Deak

**Affiliations:** Developmental Exposure Alcohol Research Center, Behavioral Neuroscience Program, Department of Psychology, Binghamton, NY, United States

**Keywords:** ethanol, miRNA, cytokine, alcohol, brain, growth factor (GF)

## Abstract

Acute ethanol exposure produces rapid alterations in neuroimmune gene expression that are both time- and cytokine-dependent. Interestingly, adolescent rats, who often consume binge-like quantities of alcohol, displayed reduced neuroimmune responses to acute ethanol challenge. However, it is not known whether growth factors, a related group of signaling factors, respond to ethanol similarly in adults and adolescents. Therefore, Experiment 1 aimed to assess the growth factor response to ethanol in both adolescents and adults. To test this, adolescent (P29–P34) and adult (P70–P80) Sprague Dawley rats of both sexes were injected with either ethanol (3.5 g/kg) or saline, and brains were harvested 3 h post-injection for assessment of growth factor, cytokine, or miRNA expression. As expected, acute ethanol challenge significantly increased IL-6 and IκBα expression in the hippocampus and amygdala, replicating our prior findings. Acute ethanol significantly decreased BDNF and increased FGF2 regardless of age condition. PDGF was unresponsive to ethanol, but showed heightened expression among adolescent males. Because recent work has focused on the PDE4 inhibitor ibudilast for treatment in alcohol use disorder, Experiment 2 tested whether ibudilast would alter ethanol-evoked gene expression changes in cytokines and growth factors in the CNS. Ibudilast (9.0 mg/kg s.c.) administration 1 h prior to ethanol had no effect on ethanol-induced changes in cytokine or growth factor changes in the hippocampus or amygdala. To further explore molecular alterations evoked by acute ethanol challenge in the adult rat hippocampus, Experiment 3 tested whether acute ethanol would change the miRNA expression profile of the dorsal hippocampus using RNASeq, which revealed a rapid suppression of 12 miRNA species 3 h after acute ethanol challenge. Of the miRNA affected by ethanol, the majority were related to inflammation or cell survival and proliferation factors, including FGF2, MAPK, NFκB, and VEGF. Overall, these findings suggest that ethanol-induced, rapid alterations in neuroimmune gene expression were (i) muted among adolescents; (ii) independent of PDE4 signaling; and (iii) accompanied by changes in several growth factors (increased FGF2, decreased BDNF). In addition, ethanol decreased expression of multiple miRNA species, suggesting a dynamic molecular profile of changes in the hippocampus within a few short hours after acute ethanol challenge. Together, these findings may provide important insight into the molecular consequences of heavy drinking in humans.

## Introduction

Alcohol exposure leads to rapid alterations in neuroimmune gene expression in the CNS (Doremus-Fitzwater and Deak, 2022). In acute exposure conditions, ethanol acts as a potent neuroimmune stimulus which mobilizes HMGB1 and potentially other ligands of toll like receptor (TLR) 4 and downstream activation of several immune signaling pathways such as NFκB and MAPK (Fernandez-Lizarbe et al., 2009; Pascual et al., 2015; Montesinos et al., 2018). TLR4 is a Pathogen Recognition Receptor (PRR) which recognizes Pathogen Associated Molecular Patterns (PAMP) and is responsible for orchestrating the proinflammatory response to TLR4 ligands such as bacterial epitopes, e.g., lipopolysaccharide (LPS) or endogenously expressed Danger-Associated Molecular Patterns (DAMPs) such as HMGB1 (Lu et al., 2008). The activation of this proinflammatory pathway in the absence of an infection, such as with ethanol exposure, is thought to produce a maladaptive neuroimmune profile (Karoly et al., 2017; Lee et al., 2018; Pascual et al., 2018). In particular, ethanol-induced MAPK enhanced transcription of proinflammatory cytokines through phosphorylation of transcription factors such as NFκB (Cuadrado and Nebreda, 2010; Sun et al., 2015). In many contexts, downstream cytokines such as IL-6, TNFα, and IL-1β serve prototypical inflammatory roles to activate phagocytic activity in microglia, and induction of apoptosis in a variety of cell types in certain conditions. Furthermore, these cytokines serve active signaling functions which modulate neuronal signaling and thereby impact behavior. For example, ethanol-induced expression of cytokines have been tied to changes in anxiety (Munshi et al., 2019a,b), learning (Hao et al., 2014), and ethanol drinking (Blednov et al., 2012; Marshall et al., 2016). It is therefore important to clarify the mechanisms by which ethanol modulates cytokine expression in the CNS, and which endogenous signaling mechanisms might work to counteract proinflammatory signaling in response to ethanol. For example, here we explore the potential role of phosphodiesterase activity in contributing to cytokine induction, and possible alterations in growth factor and/or miRNA that might moderate cytokine signaling.

Growth factor signaling often shows an effect on brain cytokine expression that is the inverse of proinflammatory cytokines such as IL-6 or IL-1β. For instance, the signaling effects of brain-derived neurotrophic factor (BDNF) are transduced through two high affinity receptors, TrkB and P75NTR, which induce downstream activation of NFκB expression, which in turn regulates BDNF expression. The behavioral and cellular effects of BDNF often have pro-growth or survival effects (Levin and Godukhin, 2017; Bruna et al., 2018; Giacobbo et al., 2018). Importantly, BDNF expression was reduced by ethanol exposure (Fernandez et al., 2017), whereas administration of exogenous BDNF was sufficient to restore LTP deficits in the hippocampus of rats in response to pyrithiamine-induced thiamine deficiency (Vedder and Savage, 2017). Likewise, in the hippocampus, exercise improved spatial memory through enhanced expression of nerve growth factor (NGF), another important growth factor in the brain (Hall et al., 2018). More recently, Fibroblast Growth Factor 2 (FGF2) has emerged as a potential target of ethanol action. Specifically, acute ethanol injection increased FGF2 expression in the hippocampus and striatum, and expression in the striatum was positively correlated to enhanced drinking phenotypes (Even-Chen et al., 2017; Even-Chen and Barak, 2019). Conversely, several studies showed that while intoxication led to increased dorsolateral striatal BDNF gene expression (Liran et al., 2020), silencing BDNF signaling in the region enhanced drinking, suggesting that endogenous BDNF may reduce or limit drinking behavior (Jeanblanc et al., 2009). Generally, high growth factor expression is protective for neurons and synapses which are liable to be degraded in an activity-dependent manner, whereas high cytokine expression increases the likelihood of cell death and phagocytosis (Turrin and Plata-Salamán, 2000; Becher et al., 2017; Zhang Y. et al., 2017).

Many studies have demonstrated that ethanol exposure during adolescence produced unique effects on brain and behavior, and implicate adolescence as a sensitive period to long-lasting effects of ethanol (Spear, 2015). Adolescence is a period of rapid neuronal development, especially in the amygdala, which is regulated by growth factor signaling (Cunningham et al., 2002; Cressman et al., 2010; Selemon, 2013; Koss et al., 2014; Riccomagno and Kolodkin, 2015; Selemon and Zecevic, 2015). Growth factors generally regulate the formation of new synapses and neurogenesis both throughout development and in adulthood (Levin and Godukhin, 2017). Overall, work in our lab and others has demonstrated that adolescent rats show blunted neuroimmune reactivity when compared to adults. Specifically, adolescent rats challenged with LPS, acute footshock challenge, or acute ethanol (i.p. or i.g.) showed substantially reduced induction of neuroimmune genes relative to adults (Doremus-Fitzwater et al., 2015; Marsland et al., 2021). These effects were observed in both the amygdala and the hippocampus, two key limbic structures that are highly sensitive to ethanol. Given the robust neuronal remodeling occurring during this time, we hypothesized that heightened adolescent growth factor expression may be one mechanism which confers lower neuroimmune reactivity among adolescents after acute ethanol challenge. This was tested in Experiment 1.

The phosphodiesterases are a class of enzymes which negatively regulate the levels of cyclic AMP (cAMP) within cells, and individual PDE subtypes are highly expressed in brain cells such as microglia and neurons (Wilson et al., 2016). For example, phosphodiesterase-4 (PDE4) inhibition is broadly associated with reduced inflammation, especially brain cytokine expression, including growth factor expression (Rolan et al., 2009). Several rodent models have demonstrated that inflammatory challenges which produced enhancements in proinflammatory cytokines such as IL-1β and TNFα were blunted with administration of PDE inhibitors (Mizuno et al., 2004; Crilly et al., 2011; Lee et al., 2012; Schwenkgrub et al., 2017; Yang et al., 2020). Furthermore, this ablation of inflammation was often accompanied by enhancements in growth factors (Mizuno et al., 2004; Schwenkgrub et al., 2017). However, it is unclear whether growth factor changes are upstream or simply concomitant with proinflammatory cytokine expression as a result of PDE inhibition. Evidence suggests that PDE4 activation may contribute to drinking behavior in rodents, given that inhibition of PDE4 was sufficient to reduce drinking in these models. In particular, in either 3 or 24 h tests of intake and preference, mice showed reduced ethanol consumption if pretreated with one of several PDE4 inhibitors (Blednov et al., 2014). In genetically alcohol preferring rats, treatment with a PDE4 inhibitor reduced ethanol consumption (Wen et al., 2012). Twice-daily injection of ibudilast, a PDE4 inhibitor, replicated this effect in two other ethanol-preferring strains of rats, and also in ethanol-dependent mice (Bell et al., 2015). In humans, treatment with ibudilast reduced the likelihood of drinking episodes among individuals diagnosed with Alcohol Use Disorder (AUD) in a clinical trial which used a dosing regimen of 20 mg b.i.d. for 2 days followed by a further 12 days of 50 mg b.i.d (Grodin et al., 2021). Halfway through this trial, subjects completed a functional MRI task which assessed brain activation evoked by visual cues associated with alcohol. Ibudilast exposure attenuated cue-induced functional connectivity between ventral striatum and the orbitofrontal cortex, anterior cingulate, medial frontal cortex, and superior frontal gyrus. In turn, the extent of the interruption of connectivity by ibudilast was predictive of heavy drinking episodes in the remaining 7 days of the trial (Burnette et al., 2021). Although the mechanisms of ibudilast action remain unclear, it is possible that PDE4 inhibition may inhibit induction of cytokines with acute ethanol exposure, which may help explain its therapeutic effects. This hypothesis was tested by treating rats with ibudilast 1 h prior to ethanol exposure, and then measuring brain cytokine mRNA responses in Experiment 2.

Micro-RNAs (miRNA) are both modulated by, and then contribute to, behavioral and physiological responses to ethanol exposure (Nunez et al., 2013; van Steenwyk et al., 2013; Mathew et al., 2016; Teppen et al., 2016). Several studies have shown dysregulated miRNA expression profiles in brains of rodents exposed to chronic ethanol regimens (van Steenwyk et al., 2013; Zhang et al., 2014; Ignacio et al., 2015; Coleman et al., 2017). For example, mice given ethanol for 5 weeks showed upregulated expression of miR-155 and miR-132 (Lippai et al., 2013). At a genome-wide level, it was demonstrated that 3 h daily drinking in a modified drinking in the dark paradigm in mice affected many of the miRNA changes shown among individuals with AUD (Nunez et al., 2013). Importantly, however, many of the changes induced by the model of early dependence produced the *opposite* effect to that observed in humans. This suggests that while acute and chronic alcohol may affect the same miRNAs, there may be a change in the direction of the regulation over the course of disease progression (Nunez et al., 2013). This picture is made more complex by recent studies showing that gene expression changes following ethanol exposure differ by cell type. After a model of escalating drinking, microglia were separated from frontal cortical tissue and changes in gene expression networks were compared to whole-brain tissue homogenate; not only were unique genes affected in microglial fractions, but nearly half of the genes which changed in both fractions were changed in opposite directions (McCarthy et al., 2018). Few studies have assessed cell-specific miRNA responses, or the total response to a first acute ethanol challenge, and this approach may highlight miRNAs that are acutely protective but become dysregulated with continued alcohol use (reviewed here: Tulisiak et al., 2017). From a functional standpoint, miRNA are a class of short non-coding RNA species, which function to inhibit mRNA translation into protein expression. This process takes place when miRNA form an RNA-induced silencing complex (RISC) with proteins which inhibit translation of an mRNA; the miRNA has a complementary sequence to the target mRNA and acts as a guide for the inhibiting proteins which then degrade the mRNA (Kobayashi and Tomari, 2016). Through these mechanisms, it is possible that early changes in miRNA may be upstream of growth factor and cytokine induction due to ethanol. Indeed, miRNA affected by ethanol exposure were often shown to target inflammatory pathways. As an example, mir-155 has been shown to exhibit proinflammatory effects in several rodent models (Lippai et al., 2013; Woodbury et al., 2015; Chen et al., 2017), likely through direct suppression of Suppressor of Cytokine Signaling 1 (SOCS1) (Nie et al., 2016). It is furthermore possible that miRNA species are induced by ethanol which could serve to limit the translation of cytokine mRNA into protein, which has been cited as a concern in recent work (Gano et al., 2019).

With these issues in mind, the current studies were designed to better understand the effect(s) of acute ethanol exposure. Specifically, Experiment 1 examined the effect of ethanol on growth factors in adolescents and adults, hypothesizing that muted induction of neuroimmune genes among adolescents might be associated with heightened growth factor expression among adolescents. Experiment 2 tested whether ibudilast, a PDE4 inhibitor that has been shown to reduce ethanol consumption in rodent models, would block the induction of neuroimmune and growth factor genes. Finally, using an unbiased RNAseq approach in Experiment 3, we assessed changes in miRNA expression 3 h after initial ethanol exposure in adult male rats, and compared differentially expressed genes to those reported in the literature for individuals with AUD as well as rodent models of ethanol exposure.

## Materials and Methods

### Subjects

For Experiments 1 and 3, male and female Sprague-Dawley rats were bred in-house from breeders originally acquired from Envigo. The day of birth was designated as P0 and litters were culled to a maximum of 10 pups per litter, with approximately equal sex ratios (*n* = 4–6 of each sex in each litter) on P1. Offspring were weaned on P21 and housed with a non-littermate until the time of experimentation. No more than 1–2 pups per litter were assigned to each experimental condition to control for litter effects (Holson and Pearce, 1992). For Experiment 2, male Sprague-Dawley rats were ordered from Envigo at a weight of 225–250 g. At the time of euthanasia rats were approximately 300 g, indicating that they had reached young adulthood. Rats were allowed to acclimate to colony conditions for 7 days before experimentation. In all cases, rats were pair-housed in standard Plexiglas^®^ bins and given free access to water and food. Each cage was provided with enrichment (wooden chew-block). The colony room was on a 12:12-h light:dark cycle (lights on at 7:00 a.m.) and kept at approximately 22°C. Rats were handled for 1–2 days prior to experimentation for identification marking and weight assessments. Rats were maintained and treated in accordance with Public Health Service (PHS) policy, and with protocols approved by the Institutional Animal Care and Use Committee at Binghamton University.

### Experimental Procedure

#### General

**FIGURE 1:**
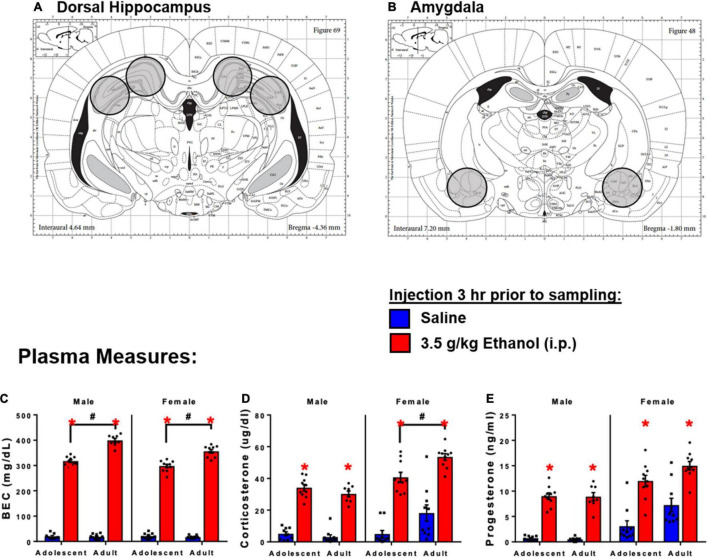
Schematic diagram of tissue sections of brain regions of interest. **(A)** Dorsal hippocampus sections. **(B)** Amygdala sections. 3 h following 3.5 g/kg ethanol, there were increases in plasma. **(C)** BECs, **(D)** CORT, and **(E)** Progesterone. Asterisk (*) and hash (#) indicate the main effect of age.

Previous work has shown that high dose (3.5–4 g/kg) acute ethanol produces rapid induction of brain cytokine expression (Doremus-Fitzwater et al., 2014; Gano et al., 2017; Gano et al., 2019), loss of righting reflex (Barney et al., 2019), and withdrawal-induced hypolocomotion (Buck et al., 2011). These doses produce BECs achieved during heavy drinking episodes in humans (300–400 mg/dl) (Usala et al., 2015; Vore et al., 2021). In the present studies, rats were exposed to ethanol through intraperitoneal (i.p.) injection of 3.5 or 4 g/kg ethanol diluted to a 20% solution in sterile, pyrogen-free physiological saline (Sigma). Injections of ethanol or vehicle occurred between 08:00 and 12:00 and brains were harvested via rapid decapitation (unanesthetized). Trunk blood was collected immediately in EDTA-coated vacutainers. Brains were extracted and snap-frozen by submersion in chilled methyl-butane on dry ice. Brains were stored at −80°C until tissue was processed for real time RT-PCR analysis (Experiments 1 and 2) or RNASeq (Experiment 3). Brain sections were harvested in a crytostat (Leica) using micro-biopsy tools (see Figure 1). Blood was centrifuged (15 min, 3,200 *g*, 4°C) for isolation of plasma.

#### Plasma Measures

Plasma was stored at −20°C and then assessed for Blood Ethanol Concentrations (BECs) using an Analox AM-1 alcohol analyzer, and for corticosterone (ADI-900-097) and progesterone (ADI-901-011) using EIA kits (Enzo Life Sciences). CORT intra-assay coefficient of variability (CV) was 1.01, and inter-assay CV was 8.93. PROG intra-assay CV was 1.20, and inter-assay CV for was 1.61. Progesterone was measured in Experiment 1 as a secondary index of neuroendocrine responses to ethanol challenge (Hueston and Deak, 2014), and its potent immunomodulatory effects (Hueston and Deak, 2020).

#### Reverse Transcription Polymerase Chain Reaction

RNA from brain tissue sections was isolated using Trizol and the commercially available RNeasy kit (Qiagen) as previously described (Doremus-Fitzwater et al., 2014). Briefly, RNA was normalized across samples using a Nanodrop spectrometer. RT-PCR was performed using SYBR-Green reagent (Qiagen) and RNA-specific primers (Table 1; IDT). For RT-PCR data, all data were adjusted to a housekeeping gene (GAPDH) and transformed using the ΔΔ*^CT^* method (Livak and Schmittgen, 2001) using the sex-specific adult vehicle condition as the control group.

**TABLE 1:** Primer sequences for genes of interest in Experiments 1 and 2.

Gene	Forward/Reverse	Accession number
GAPDH	GTGCCAGCCTCGTCTCATAG AGAGAAGGCAGCCCTGGTAA	NM_017008
IL-6	TAGTCCTTCCTACCCCAACTTCC TTGGTCCTTAGCCACTCCTTC	NM_012589.2
IκBα	CTGTTGAAGTGTGGGGCTGA AGGGCAACTCATCTTCCGTG	NM_001105720.2
FGF2	GAGCGACCCACACGTCAA TTCCGTGACCGGTAAGTGTT	NM_019305.2
BDNF	TGGCAGGCTTTGATGAGACC CAGCCTTCATGCAACCGAAG	NM_001270630.1
PDGFβ	AGACAGTAGTGACCCCTCGG TAAATAACCCTGCCCACACTCT	NM_031524.1
NGF	CATCGCTCTCCTTCACAGAGTT TGTACGGTTCTGCCTGTACG	NM_001277055.1
VEGFa	CTGTACCTCCACCATGCCAA AACTTCACCACTTCATGGGCT	NM_001110333.2

*Gene accession number is listed.*

#### Experiment 1

The goal of Experiment 1 was to assess the expression of growth factors in adults and adolescents under basal and ethanol-stimulated conditions. We hypothesized that growth factors may be upregulated in adolescents compared to adults, and that growth factors would show acute responses to ethanol. This was tested using a 2 (sex) × 2 (age) × 2 (ethanol) factorial design. Male and female rats (*n* = 10 per group, *N* = 80) were injected with 3.5 g/kg (i.p.) ethanol or saline during early adolescence (P32–33) or during young adulthood (P77–87), euthanized 3 h later, and brains were harvested as described above.

#### Experiment 2

Phosphodiesterase-4 inhibitors are under study for potential benefits in reducing drinking among individuals with AUD (Blednov et al., 2014; Logrip, 2015; Ray et al., 2017; Grodin et al., 2021). We aimed to assess the effect of PDE4 modulation of ethanol-related neuroimmune and growth factor genes, given the importance of these targets in modulating drinking behaviors. To test the potential modulation of ethanol-induced cytokine mRNA expression by PDE4 inhibition in the brain, we injected ibudilast prior to ethanol exposure in a 2 (ibudilast) × 2 (ethanol) factorial design. Adult male Sprague Dawley rats (*n* = 10 per group, *N* = 40) were injected with ibudilast (9 mg/kg s.c.) 1 h before ethanol was administered. This dose of ibudilast was chosen because it was shown to reduce ethanol drinking in mice when given s.c. (Bell et al., 2015). Ethanol (3.5 g/kg) was administered i.p. and rats were euthanized 3 h later. RNA extraction and RT-PCR were performed as described above. Based on the results of Experiment 1, analyses of both plasma hormones and target genes were narrowed to focus only on factors that were significantly affected by ethanol.

#### Experiment 3

miRNA have emerged as important biomarker targets to assess the impact of ethanol on the brain. Ethanol exposure changes expression of miRNAs in the brain in unique ways depending on the ethanol exposure procedure (Nunez et al., 2013; van Steenwyk et al., 2013; Mathew et al., 2016; Teppen et al., 2016; Coleman et al., 2017). miRNA are responsible for silencing the translation of mRNA into protein after transcription takes place (Kobayashi and Tomari, 2016). However, the interaction between miRNA expression and cytokine expression is less clear. Therefore, we assayed the effect of acute ethanol exposure on hippocampal miRNA expression, to identify potential modulators of cytokine mRNA induction and translation to protein. Adult male Sprague Dawley rats (*n* = 8 per group, *N* = 16) were injected with ethanol (4.0 g/kg) in early adulthood (P70–80) and euthanized 3 h later. This higher dose was used based on prior work from our lab examining behavioral (Richey et al., 2012; Barney et al., 2019), neuroendocrine (Buck et al., 2011), and rapid alterations in neuroimmune gene expression were evident in the 3.5–4.0 g/kg dose range (Doremus-Fitzwater et al., 2014; Gano et al., 2017). Brain tissue sections were submitted to RNAseq including enrichment for miRNA in the sample similar to that used previously (Ignacio et al., 2014). For each rat, 1 μg of total RNA was purified using the TruSeq Small RNA Sample Prep kit (Illumina, San Diego, CA, United States) and subsequent gel purification for small (20–30 nt) RNA species. Libraries were indexed and multiplexed in sets of 8 (6 sets total) prior to sequencing (single-end, 37 cycles) using Reagent Kit v3 reagents on a MiSeq Benchtop Sequencer (Illumina, San Diego, CA, United States). Any species showing fewer than 10 reads in 10 samples were trimmed from the study. Data were normalized to median expression level per group, and then mean centered. These procedures and the following fold change and *t-*test analysis were completed using Metaboanalyst online analysis software.

### Statistics

#### Experiment 1 and 2

Omnibus ANOVAs were performed for all dependent measures using ANOVA designs that mirrored the experimental designs described above. Data were compared using 2 × 2 ANOVA with separate analyses run for males and females where appropriate. Tukey’s HSD tests were used as *post-hoc* to follow up on significant main effects and interactions, α was set at 0.05. Two samples were omitted from all measures in the adult male ethanol group in Experiment 1 due to experimenter error. In Experiment 2, one sample in the amygdala was removed from all PCR measures due to insufficient RNA extraction.

#### Experiment 3

*t*-tests comparing expression of miRNA between groups were used. Raw *p* values were used to rank differentially expressed miRNA of interest, and were shown in Figure 5.

## Results

### Experiment 1

#### Plasma Measures

Injection of ethanol yielded higher BECs in adults compared to adolescents in both males [Interaction: *F*_(1,33)_ = 66.6, *p* < 0.05] and females [Interaction: *F*_(1,36)_ = 34.9] (Figure 1C). Ethanol increased corticosterone in males [Main effect: *F*_(1,33)_ = 290, *p* < 0.05] and females [Main effect: *F*_(1,36)_ = 112, *p* < 0.05]. There was also a main effect of age on CORT in females [*F*_(1,36)_ = 15.1, *p* < 0.05], and adolescent females showed lower corticosterone at the 3 h time point (*p* < 0.05) (Figure 1D). Ethanol increased progesterone in males [Main effect: *F*_(1,32)_ = 270, *p* < 0.05] and females [Main effect: *F*_(1,36)_ = 57.7, *p* < 0.05]; no differences between adolescents and adults were observed (Figure 1E).

#### Hippocampus

**FIGURE 2:**
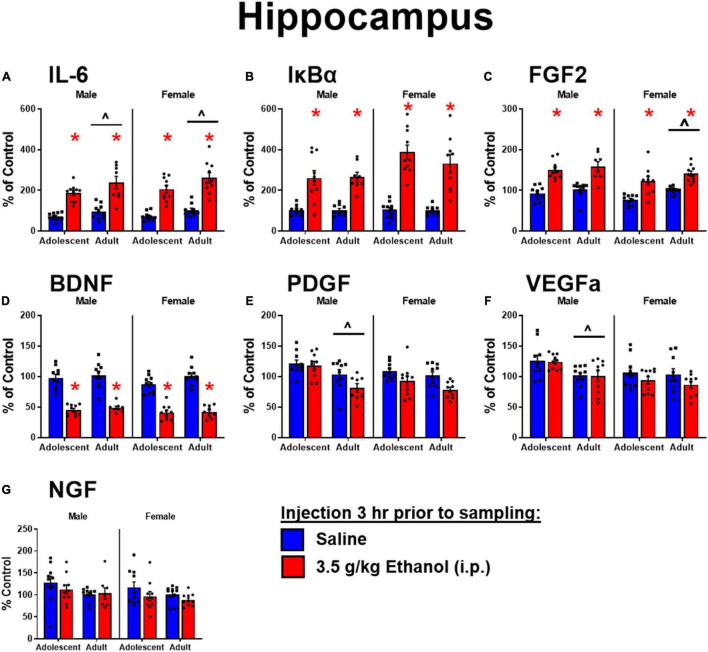
Ethanol-induced gene expression in adolescents and adults. Gene expression of IL-6 **(A)**, Iκ Bα **(B)**, FGF2 **(C)**, BDNF **(D)**, PDGF **(E)**, VEGFα **(F)**, and NGF **(G)** in the dorsal hippocampus were assessed in ratsi.p. injection of saline or 3.5 g/kg ethanol. Gene expression data were calculated as a relative change in gene expression using the 2-ΔΔC(t) method, with (GAPDH) used as a reference gene and the vehicle – saline group serving as the ultimate control for each sex individually. Bars denote group means ± standard error of the mean (represented by vertical error bars). Asterisks (*) signify significant ethanol-induced changes (*p* < 0.05), chevron (^∧^) indicates main effect of age.

Ethanol increased IL-6 in both males [main effect of ethanol: *F*_(1,34)_ = 66, *p* < 0.05] and females [main effect of ethanol: *F*_(1,36)_ = 80, *p* < 0.05] regardless of age, although adolescents had lower levels of IL-6 overall [main effect of age: *F*_(1,34)_ = 5.3; *F*_(1,36)_ = 7.09] (Figure 2A). Ethanol increased IκBα expression in males [main effect of ethanol: *F*_(1,34)_ = 53, *p* < 0.05] and females [main effect of ethanol: *F*_(1,35)_ = 80.1, *p* < 0.05], while there was no effect of age (Figure 2B). Ethanol increased FGF2 in both males [main effect of ethanol: *F*_(1,34)_ = 56.9, *p* < 0.05] and females [main effect of ethanol: *F*_(1,36)_ = 42.6, *p* < 0.05] regardless of age, but female adolescents showed less FGF2 regardless of ethanol condition [main effect of age: *F*_(1,36)_ = 9.67, *p* < 0.05] (Figure 2C). Ethanol decreased BDNF in both males [main effect of ethanol: *F*_(1,34)_ = 115, *p* < 0.05] and females [main effect of ethanol: *F*_(1,36)_ = 166, *p* < 0.05] regardless of age (Figure 2D). PDGF was higher in adolescent males [main effect of age: *F*_(1,34)_ = 15.8, *p* < 0.05] regardless of ethanol, an effect that seems to be driven by decreases seen only in adults given ethanol, although no significant interaction was detected. In females, PDGF was decreased after ethanol [main effect of ethanol: *F*_(1,36)_ = 11.2, *p* < 0.05], an effect likely driven by the change in adults given ethanol (n.s. comparison between adults and adolescents with ethanol) (Figure 2E). VEGFa was higher in adolescents for males only [main effect of age: *F*_(1,34)_ = 13.7, *p* < 0.05] regardless of ethanol exposure (Figure 2F). NGF was unaffected by ethanol or age in both males and females (Figure 2G).

#### Amygdala

**FIGURE 3:**
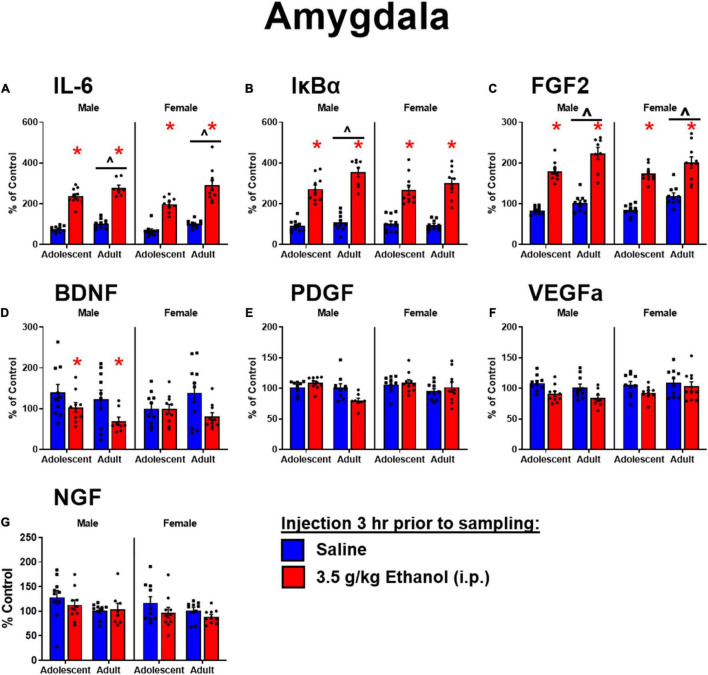
Ethanol-induced gene expression in adolescents and adults. Gene expression of IL-6 **(A)**, IκBα **(B)**, FGF2 **(C)**, BDNF **(D)**, PDGF **(E)**, VEGFα **(F)**, and NGF **(G)** in the amygdala were assessed in rats i.p. injection of saline or 3.5 g/kg ethanol. Gene expression data were calculated as a relative change in gene expression using the 2-ΔΔC(t) method, with (GAPDH) used as a reference gene and the vehicle – saline group serving as the ultimate control for each sex individually. Bars denote group means ± standard error of the mean (represented by vertical error bars). Asterisks (*) signify significant ethanol-induced changes (*p* < 0.05), chevron (^∧^) indicates main effect of age.

Ethanol increased IL-6 in both males [main effect of ethanol: *F*_(1,34)_ = 288, *p* < 0.05] and females [main effect of ethanol: *F*_(1,36)_ = 111] regardless of age. IL-6 was globally lower in adolescent males [main effect of age: *F*_(1,34)_ = 11] and females [*F*_(1,36)_ = 17.2] (Figure 3A). Ethanol increased IκBα in both males [main effect of ethanol: *F*_(1,34)_ = 166, *p* < 0.05] and females [main effect of ethanol: *F*_(1,36)_ = 105, *p* < 0.05]. IκBα was lower in adolescent males [main effect of age: *F*_(1,34)_ = 8.7, *p* < 0.05] but not females (Figure 3B). Ethanol increased FGF2 in both males [main effect of ethanol: *F*_(1,34)_ = 175, *p* < 0.05] and females [main effect of ethanol: *F*_(1,36)_ = 94.4, *p* < 0.05] regardless of age, while overall adolescents showed lower FGF2 levels in males [main effect of age: *F*_(1,34)_ = 14, *p* < 0.05] and females [main effect of age: *F*_(1,36)_ = 12.6, *p* < 0.05] (Figure 3C). Ethanol decreased BDNF in males [main effect of ethanol: *F*_(1,34)_ = 6.9, *p* < 0.05] but not females: observed decreases in BDNF following ethanol were not significant in females likely due to a smaller effect size among adolescents (Figure 3D). PDGF in males showed an interaction of age and ethanol [interaction term: *F*_(1,34)_ = 11, *p* < 0.05] driven by a decrease in expression only in adults exposed to ethanol (*p* < 0.05). Regardless of ethanol, adolescent males showed higher PDGF expression [main effect of age: *F*_(1,34)_ = 11, *p* < 0.05]. Females however showed no main effects or interactions in PDGF expression (Figure 3E). A similar pattern to BDNF was observed for VEGFa, with an effect of ethanol to decrease expression only seen in males [main effect of ethanol: *F*_(1,34)_ = 13, *p* < 0.05] (Figure 3F). NGF was increased with ethanol exposure in males [main effect of ethanol: *F*_(1,34)_ = 10] driven by the effect in adolescents, and females [main effect of ethanol: *F*_(1,36)_ = 7.73, *p* < 0.05]. In females, NGF was higher in adolescents overall [main effect of age: *F*_(1,36)_ = 8.48, *p* < 0.05] driven by enhanced expression in adolescents exposed to ethanol compared to their vehicle counterparts (*p* < 0.05) (Figure 3G).

### Experiment 2

#### Plasma Measures

**FIGURE 4:**
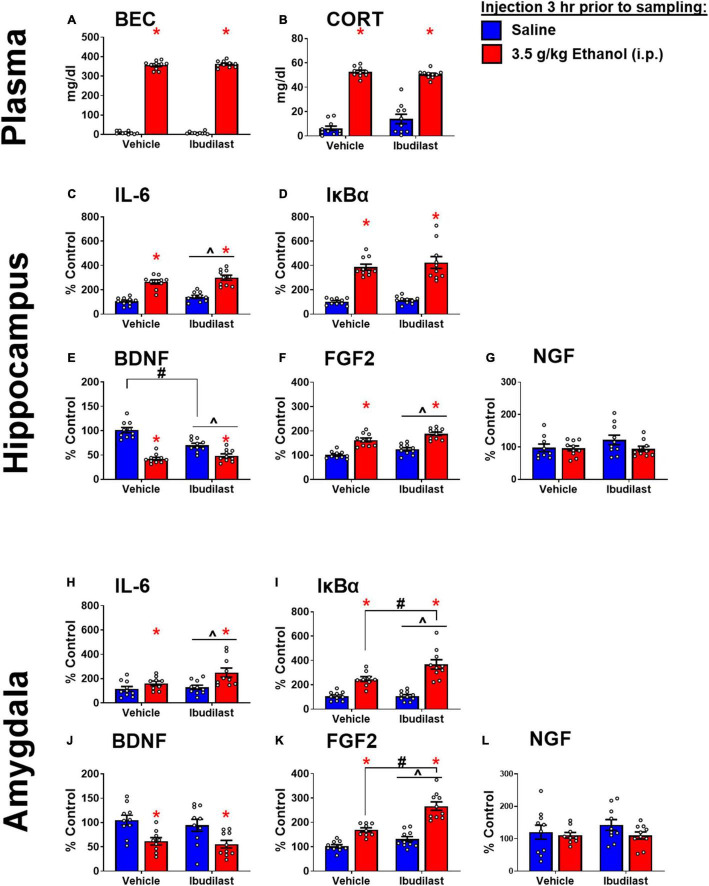
Ethanol-induced gene expression in adult males treated with ibudilast. **(A)** BECs, **(B)** CORT levels in plasma, gene expression of IL-6 **(C,H)**, IκBα **(D,I)**, BDNF **(E,J)**, FGF2 **(F,K)**, and NGF **(G,L)** in the dorsal hippocampus and amygdala were assessed in rats pretreated with ibudilast or vehicle before i.p. injection of saline or 3.5 g/kg ethanol. Gene expression data were calculated as a relative change in gene expression using the 2-ΔΔC(t) method, with (GAPDH) used as a reference gene and the vehicle – saline group serving as the ultimate control for each sex individually. Bars denote group means ± standard error of the mean (represented by vertical error bars). There is a main effect of ethanol in every measure. Chevron (^∧^) indicates main effect of ibudilast, asterisk (*) indicate significant interaction of ethanol and ibudilast with significant posthoc comparison (Tukey’s).

Similar to Experiment 1, ethanol injection yielded BECs of approximately 350 mg/dl, regardless of ibudilast administration (Figure 4A). There was a main effect of ethanol to increase CORT [*F*_(1,36)_ = 325, *p* < 0.05]. There was also an interaction between ibudilast and ethanol [*F*_(1,36)_ = 4.42, *p* < 0.05], but there were no significant *post-hoc* comparisons (Figure 4B). The interaction appears to be driven by a slight increase in CORT among rats exposed to ibudilast compared to vehicle, but no effect of ibudilast to enhance the CORT response to ethanol. There was no main effect of ibudilast on CORT [*F*_(1,36)_ = 1.47, *p* = 0.2326]. Progesterone was not assessed in this experiment.

#### Hippocampus

Main effects of both ethanol [*F*_(1,36)_ = 107, *p* < 0.05] and ibudilast [*F*_(1,36)_ = 5.63, *p* < 0.05] to increase IL-6 expression were observed, but there was no significant interaction [*F*_(1,36)_ = 0.00815, *p* = 0.93] (Figure 4C). Ethanol increased IκBα expression [main effect of ethanol: *F*_(1, 36)_ = 114, *p* < 0.05], while there was no effect of ibudilast [main effect of ibudilast: *F*_(1,36)_ = 0.8, *p* = 0.38] (Figure 4D). There was a significant interaction between ethanol and ibudilast administration to decrease BDNF expression [interaction term: *F*_(1,36)_ = 20, *p* < 0.05], such that ibudilast and ethanol together group had lower expression than the ibudilast-only group (*p* < 0.05) (Figure 4E). Both ethanol [main effect of ethanol: *F*_(1,36)_ = 84.6, *p* < 0.05] and ibudilast [main effect of ibudilast: *F*_(1,36)_ = 12.9, *p* < 0.05] increased expression of FGF2, but no interaction was observed (Figure 4F). There were no significant effects of either drug on NGF expression (Figure 4G).

#### Amygdala

Main effects of both ethanol [main effect of ethanol: *F*_(1,35)_ = 11.3, *p* < 0.05] and ibudilast [main effect of ibudilast: *F*_(1,35)_ = 4.13, *p* < 0.05] to increase IL-6 expression were observed, but there was no significant interaction [*F*_(1,35)_ = 2.35, *p* = 0.13] (Figure 4H). Ethanol increased IκBα expression [main effect of ethanol: *F*_(1,35)_ = 73.2, *p* < 0.05], as did ibudilast [main effect of ibudilast: *F*_(1,35)_ = 6.46, *p* < 0.05], and ibudilast potentiated the effect of ethanol (*p* < 0.05) (Figure 4I). There was a significant main effect of ethanol to decrease BDNF expression [main effect of ethanol: *F*_(1,35)_ = 17.7, *p* < 0.05], but there was no effect of ibudilast [*F*_(1,35)_ = 0.711, *p* = 0.40] (Figure 4J). Ethanol and ibudilast interacted to increase expression of FGF2 [interaction term: *F*_(1,35)_ = 8.55, *p* < 0.05], such that ethanol and ibudilast together increased expression more than ethanol alone (*p* < 0.05) (Figure 4K). There were no significant effects of either stimulus on NGF expression (Figure 4L).

### Experiment 3

#### Plasma Measures

**FIGURE 5:**
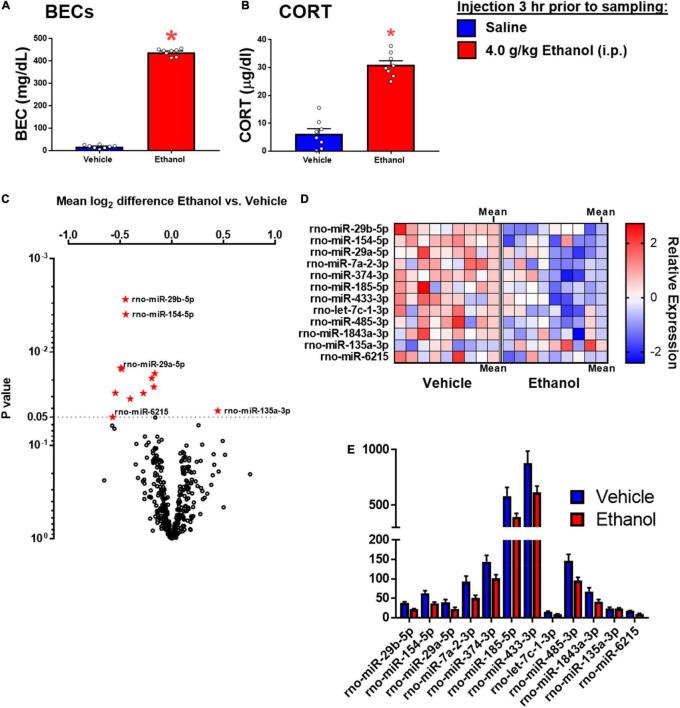
Ethanol-induced miRNA expression in adult males 3 h after ethanol. **(A)** Blood ethanol concentrations at 3 h after 4 g/kg ethanol, and **(B)** Plasma corticosterone were both increased. **(C)** Volcano plot for miRNA expression in the hippocampi of rats given saline or ethanol injection 3 before tissue collection. The miRNA which were significantly affected are shown in panel **(D)**. **(D)** A heat map which shows individual sample changes in expression level for miRNA identified in panel **(C)**, and mean for each group in the last column of each group. **(E)** Raw expression values for each miRNA significantly affected in panel **(A)**. Asterisk (*) indicates the effect of ethanol (t-test).

Ethanol injection yielded BECs of approximately 430 mg/dl, commensurate with the 4 g/kg dose used in this experiment (Figure 5A). There was a significant effect of ethanol to increase CORT [*t*_(1,14)_ = 10.45, *p* < 0.05] (Figure 5B).

#### miRNA in the Dorsal Hippocampus

Fold change and *t-*test analysis revealed that 12 miRNAs were significantly affected by ethanol exposure (raw *p* < 0.05). No corrections for multiple tests were used in this exploratory analysis. Of the miRNA affected by ethanol, all but one miRNA were suppressed by ethanol, with only one miRNA species (miR-135a-3p) showing enhancement due to ethanol. Figure 5A uses a volcano plot to illustrate the effect of ethanol by plotting the *p*-value against the fold-change, whereas Figure 5B shows a heatmap indicating expression patterns of the 12 genes significantly affected by ethanol. Note that relatively moderate fold changes in miRNA were observed (0.7–1.3 fold), suggesting only a modest influence of acute ethanol challenge on miRNA expression in the dorsal hippocampus. This is highlighted by Figure 5E, which shows raw expression values for each of the ethanol-affected miRNA, and also shows the wide variance in expression levels across miRNA. Many of the miRNAs affected have been linked to cell proliferation/survival and inflammation (Table 2). The unbiased miRNA database for predicted targets miRDB was used to generate *in silico* targets of these miRNA with predicted homology scores of above 90%. In many cases, there were more than five targets above 90%, but were not listed. PUBMED searches including terms “ethanol” and/or “alcohol,” “brain” and the miRNA of interest (not species-specific; i.e., miR-129a-5p instead of rno-miR-129a-5p) were used to select the most relevant article(s) for review in Table 2. In some cases, no articles including all three terms were found, in which case “brain” and/or “ethanol/alcohol” were dropped from the search string. Only one miRNA returned no relevant articles under these search parameters.

**TABLE 2:** Differentially expressed miRNA in Experiment 3, using a raw *p*-value of <0.05.

DEmiRNA	*p*	Top five predicted (miRDB – score > 90)	Effect in the literature	References
mir-29b-5p	0.003	Elavl2, Nr4a3, Herc3, Mettl15, and Ppp1r2	Overexpression of 29b reduced neuronal expression of NET and glucocorticoid receptors *in vitro*. Overexpression reduced STAT3 activation *in vitro*, and KO *in vivo* enhanced inflammation in the liver.	Deng et al., 2016; Zhou et al., 2022
mir-154-5p	0.004	Rbm34, Cdca4, Ube3a, Hnrnpr, and Trem3	154-5p expression upregulation in the brain reversed development of neuropathic pain, and reduced levels of cytokine expression in microglia of rats. 154-5p directly targeted AQP9	Wu et al., 2020
mir-7a-2-3p	0.015	Klf3, Cav1, Serinc5, Olig5, and Arpc2	In a model of hypoxic-ischemia in neonatal rats 7a-2-3p was decreased in the brain. *In vitro* assays demonstrated that overexpression of 7a-2-3p increased survivability of neurons and reduced apoptosis, possibly through silencing expression of Vimentin	Zhang et al., 2019
mir-185-5p	0.019	Synm, Slc8a1, Ankfy1, Sgms1, and Eya3	185-5p directly silenced VEGFA to reduce angiogenesis induced by injection of dehydroepiandrosterone in rats or VEGF administration in human endothelial cells	Wei and Zhao, 2020
let-7c-1-3p	0.027	Stag1, Cdh11, Slain2, Atp5md, and Nampt	let-7c-1-3p was enhanced specifically in M2 macrophages	Lu et al., 2016
mir-485-3p	0.028	Xpo4, Slc6a19, Trappc6b, Naa15, and Fez1	485-3p overexpression enhanced neural stem cell differentiation into neurons and inhibited proliferation of cells, achieving both through silencing of TRIP6, in mice	Gu et al., 2020
mir-6215	0.050	Plagl1, Tmem35a, Alox12, Arcn1, and f6	Bone samples taken after induction of bone deterioration with antimetabolite methotrexate showed enhanced expression of miR6215 in rats	Zhang Y. et al., 2021
miR-29a-5p	0.015	Trak2, Eng, Lrrtm3, Ms4a2, and Ttc8	Directly targeted NLRP inflammasome, resulting in reduced severity of BBB permeability changes due to TBI, and increased levels of IL-1β. *In vitro*, reduced NLRP activation of macrophages.	Zhang A. et al., 2021; Zhang M. L. et al., 2021
miR-374-3p	0.017	Atad1, Slc6a8, Sparc, Has3, and Slc8a1	Targeted WNT5B *in vitro* to reduce MAPK-associated gene expression, resulting in lower LPS-induced inflammation in cells.	Shi and Ren, 2020
miR-433-3p	0.024	Mapk8, Azin1, Plvap, Oma1, and Cox18	Reduced cell proliferation *in vitro*. Indirectly reduced FGF2 expression *in vitro* through competition. *In vitro*, can directly target TLR10 to modulate NFκB activation	Sun et al., 2017; Cai et al., 2020
miR-1843a-3p	0.032	Ndc1 and Slamf6	No relevant articles	
miR-135a-3p	0.043	Synemin, Vps35, Srd5a, Slc39a12, and Snrpn	Being investigated for role in cancer, especially ovarian, as it suppressed tumor growth through targeting CCR2 gene. *In vivo* and *in vitro*, expression of miRNA reduced angiogenesis induced by injury or VEGF application.	Duan et al., 2018; Icli et al., 2019; Ghafouri-Fard et al., 2020

*Relevant articles were briefly summarized and cited. miRDB database was used to identify in silico target of affected miRNA, with at least 90% homology of the target mRNA sequence.*

## Discussion

In these experiments, we examined the relationship between cytokine, growth factor, and miRNA expression profiles after a binge-like ethanol exposure, focusing on the period of high intoxication (3 h post injection). This ethanol exposure procedure was used to model high-intensity drinking, which is a subset of drinking behavior that is especially prevalent among adolescents and individuals with AUD (Patrick and Terry-McElrath, 2017; Chung et al., 2018). This model is especially useful for gaining insight into molecular changes produced by ethanol, before homeostatic changes or tolerance have taken place with repeated exposure. We then assessed the potential ability of ibudilast to modulate ethanol-induced cytokine signaling. Overall, we observed rapid, acute changes in neuroimmune genes that largely replicated findings we have observed in numerous prior studies (Doremus-Fitzwater et al., 2014, 2015; Gano et al., 2017; Vore et al., 2021), supporting the replicability of these effects. In addition, we add new findings on the expression of multiple growth factors in adult males, and an evaluation of whether these effects were comparable between adolescents and adults for growth factor expression.

In Experiment 1, acute ethanol exposure enhanced the expression of IκBα and IL-6 in the hippocampus and amygdala consistent with activation of the NFκB pathway and our previous work. Our previous work has also indicated that the neuroimmune system is relatively immature during adolescence in the rat, evidenced by reduced basal and evoked expression of neuroimmune genes (Doremus-Fitzwater et al., 2015; Marsland et al., 2021). Although in some instances such as IL-6 in the hippocampus following 4 g/kg ethanol these effects manifested as interactions between ethanol administration and age, many of these age-related differences manifested as simple main effects of age. For example, in the amygdala of the same rats, there were main effects of both age and ethanol (Doremus-Fitzwater et al., 2015). The current data from Experiment 1 support the interpretation that adolescents have lower basal expression of some inflammatory factors in the brain, given that there were main effects of age in the amygdala and hippocampus for IL-6. However, it must be noted that this was not a ubiquitous phenomenon. For example, IκBα did not follow this pattern in the hippocampus. Although our prior work has shown few if any sex differences in ethanol-induced changes in neuroimmune gene expression (Gano et al., 2017, 2019), this is the first demonstration that expression of neuroimmune genes was muted in adolescent females as well. Sex differences in the expression of neuroimmune proteins in the brain have also been observed; adult females had greater induction of IL-1β with probe insertion, whereas males had greater increases in chemokine, especially CCL3, as measured by large-molecule microdialysis (Perkins et al., 2021). While the magnitude of ethanol-induced changes were much greater than differences driven by age, lower levels of cytokine (basal or induced) has important implications for ongoing development of the brain during this period. This is highlighted by studies showing long-lasting reductions in healthy synapses in adults due to adolescent inflammatory challenge (Risher et al., 2015; Pascual et al., 2018). Thus, the unique profile of immune function in the adolescent may provide a mechanism by which ethanol influences the trajectory of neurobehavioral development more robustly during this period.

Of the growth factors examined here, certain effects stand out as particularly interesting. For instance, FGF2 was increased after acute ethanol in both the hippocampus and amygdala regardless of age, although to a greater extent in the amygdala than the hippocampus. Furthermore, this growth factor was less expressed (main effect) in the amygdala of adolescents compared to adults. These findings are important given studies showing that FGF2 signaling enhanced the drinking behavior of rodents (Even-Chen et al., 2017; Even-Chen and Barak, 2019), suggesting that FGF2 could serve as a target for curbing relapse drinking, specifically in the dorsomedial striatum. Other work has focused on the effects of FGF2 on anxiety and shown that endogenous FGF2 in the hippocampus reduced anxiety behavior in rats (Perez et al., 2009; Eren-Koçak et al., 2011). This is especially notable given the relatively high brain expression of FGF2 among individuals with mood disorders (Evans et al., 2004). Therefore, enhanced FGF2 expression observed in the amygdala should motivate studies of FGF2 signaling in the context of ethanol modulation of anxiety-like behaviors. Furthermore, our data show an acute downregulation of BDNF in both regions when intoxicated, which is consistent with previous research showing that ethanol exposure reduced BDNF (Fernandez et al., 2016; Fernandez et al., 2017). These changes were related to adult impairments in hippocampal-dependent behaviors (Fernandez et al., 2017). Finally, VEGFa was significantly higher in adolescents only in males, and was unaffected by ethanol exposure. This is an interesting observation given the recent tie between VEGFa expression following adolescent intermittent ethanol exposure and male-specific increases in blood brain barrier (BBB) permeability seen after this exposure paradigm (Vore et al., 2022). These data support the hypothesis that the male adolescent BBB may be uniquely susceptible to alcohol perturbation because of higher basal VEGFa activity, although a mechanistic test of this has yet to be performed.

Ibudilast has been shown to reduce ethanol drinking in rodent models (Bell et al., 2015) and is currently under clinical study for use among individuals with AUD due to its recognized anti-inflammatory, pro-growth factor profile (Ray et al., 2017; Grodin et al., 2021). In mixed microglia and neuronal cell culture, administration of LPS enhanced activity of microglia resulting in greater neuronal cell death (Mizuno et al., 2004). Ibudilast treatment rescued much of the LPS-induced cell death, and produced modest reductions in IL-6 and IL-1β while it increased NGF (Mizuno et al., 2004). A similar neuroprotective role of ibudilast was observed with repeated ibudilast treatment prior to MPTP-induced neuronal death in the striatum, accompanied by increased GDNF (Schwenkgrub et al., 2017). However, it is unknown how acute ibudilast and high dose ethanol interact on cytokine and growth factor signaling. As a start, we showed here that, contrary to our expectations, ibudilast did not reverse the effects of ethanol on neuroimmune or grown factor gene expression as we had predicted. In contrast, ibudilast produced small effects on IL-6, BDNF, and FGF2 in the same direction as ethanol in the hippocampus, and seemed to potentiate most effects of ethanol in the amygdala. Although these effects would seem to conflict with the extant literature showing that ibudilast reduced the pro-inflammatory profile in the brain (Lee et al., 2012; Schwenkgrub et al., 2017; Yang et al., 2020), it should be noted that the neuroimmune gene expression profile evoked by experimenter-administered ethanol also shares characteristic anti-inflammatory suppression of TNFα and IL-1β, similar to anti-inflammatory glucocorticoid exposure (Barnum et al., 2008; Vore et al., 2017). It should also be noted that only a single dose of ibudilast was tested against a single high dose of ethanol exposure, so it may be prudent to conduct more elaborate dose-response functions in future studies. It is also possible that chronic exposure to ethanol and/or ibudilast, typically twice daily for weeks, may have categorically different effects than acute injection of the drugs alone or in combination (Ray et al., 2017; Grodin et al., 2021). These parametric experiments have not been completed to our knowledge, but could offer one route to increasing the therapeutic potential of ibudilast for the treatment of AUD.

Acute ethanol exposure produced changes in expression levels of 12 miRNA species. Overall, we observed suppression of these miRNA in the hippocampus of rats euthanized at 3 h after ethanol. Although the mechanism of this suppression is currently unknown, a general suppression of miRNA could be the result of ethanol-induced impairments in DICER machinery, or other processes involved in the production of the mature miRNA species of interest. Future studies should assess both immature, longer miRNA species, and the function of DICER processes and their modulation by ethanol exposure. Of the miRNA most affected by ethanol (shown in Table 2), many are known to target signaling molecules or downstream pathways assessed in Experiments 1 and 2. Some of the affected miRNA species can directly silence cytokines and growth factors; mir-185-5p inhibited VEGFa (Wei and Zhao, 2020), whereas 31-5p targeted IL-34 (Oshima et al., 2021). Recently, it was shown that the miRNA affected here with the lowest *p* value, miR-29b, directly targeted STAT3, thereby controlling inflammatory cytokine expression in the liver, and reduced expression in macrophages *in vitro* (Zhou et al., 2022). At 3 h after high-dose ethanol, the downregulation of this miRNA may contribute to the induction of cytokines through upstream disinhibition of STAT3 signaling. Overall, many of the affected miRNA here have emerged from the oncology field, where several miRNA have shown an ability to change tumor growth and cell differentiation through interactions with inflammatory signaling pathways (Table 2; Saad et al., 2015; Cai et al., 2018). This could have relevance for the long-standing association between alcohol misuse and cancer prevalence rates (Marziliano et al., 2020; Rehm et al., 2020).

Although the miRNA most affected by acute ethanol exposure have not been directly tied to ethanol-related behaviors yet, several studies have identified a handful of miRNA that are functionally important. For example, mir-494 inhibition through targeted infusion into the central amygdala reduced anxiety-like behavior similar to 1 g/kg ethanol i.p. Importantly, this miRNA was downregulated by the same dose of ethanol when amygdala was collected immediately after behavioral testing (Teppen et al., 2016). However, in the RNASeq analysis used in the present experiment, copy number of mir-494 was below 10 per rat, highlighting two elements of our analytical approach: (1) the functional importance of miRNA that exist at relatively low expression levels under basal and stimulated conditions, and (2) the consideration of read-depth for RNAseq analyses especially in the case of these low-abundancy miRNA. With this in mind, several unresolved issues from this preliminary miRNA study should be addressed with future studies. First, future studies should include a direct comparison of adolescent and adult miRNA signatures including both males and females, and consider the possibility of comparing whole tissue punches (i.e., mixed populations of cells) to cell-type specific extractions. Likewise, a limitation of Experiment 2 was that females were not included, and the effects of PDE manipulations in females should be assessed. Second, the effects reported here should be validated using secondary strategies, such as PCR, to strengthen confidence in the findings observed. Finally, future studies could attempt to restore miRNAs of interest and tie these effects to functional outcomes associated with acute ethanol exposure.

In humans, alcohol use and misuse often begin during adolescence, and it seems that dependence and repeated exposure modulates effects of ibudilast. Likewise, repeated exposure to ethanol changes the function of GABA and cytokine signaling, in an interdependent manner. Recent research has shown that cytokine signaling induced by ethanol exposure modulated GABAergic signaling in the central amygdala (Patel et al., 2021). These changes were seen in adults, but changes in GABA signaling have also been seen after ethanol in adolescents. For example, adolescent alcohol exposure decreased δ and α4 subunit expression into adulthood in the hippocampus, while the same exposure given during adulthood did not produce any changes in GABA receptors (Centanni et al., 2014). An *in vitro* experiment demonstrated that in neuronal cultures, application of TNFα caused internalization of GABA receptors in neurons, decreasing total available GABA receptors on the surface of the cell (Stellwagen et al., 2005). Chronic ethanol exposure in adult mice enhanced expression of IL-1β in neurons in the central amygdala, and IL-1β was shown to directly affect GABAergic signaling (Vezzani and Viviani, 2015; Patel et al., 2019). Future research should further explore how adolescent ethanol exposure affects cytokine signaling, and how the unique neuroimmune profile in adolescence may modulate effects of cytokines on neuronal signaling.

## Data Availability Statement

The datasets presented in this study can be found in online repositories. The name of the repository and accession number can be found below: https://www.ncbi.nlm.nih.gov/geo/, Gene Expression Omnibus and Sequence Read Archive under the series accession number GSE199848.

## Ethics Statement

The animal study was reviewed and approved by IACUC Binghamton University.

## Author Contributions

TB: conceptualization, methodology, validation, formal analysis, investigation, writing−original draft, review, and editing, visualization, and supervision. AV: conceptualization, methodology, investigation, review and editing, and supervision. TD: conceptualization, methodology, writing−review and editing, supervision, project administration, and funding acquisition. All authors contributed to the article and approved the submitted version.

## Author Disclaimer

Any opinions, findings, and conclusions or recommendations expressed in this material are those of the author(s) and do not necessarily reflect the views of the above stated funding agencies.

## Conflict of Interest

The authors declare that the research was conducted in the absence of any commercial or financial relationships that could be construed as a potential conflict of interest.

## Publisher’s Note

All claims expressed in this article are solely those of the authors and do not necessarily represent those of their affiliated organizations, or those of the publisher, the editors and the reviewers. Any product that may be evaluated in this article, or claim that may be made by its manufacturer, is not guaranteed or endorsed by the publisher.
